# Evaluation of Biointegration and Inflammatory Response to Blood Vessels Produced by Tissue Engineering—Experimental Model in Rabbits

**DOI:** 10.3390/biom12121776

**Published:** 2022-11-29

**Authors:** Mariana Thaís Silva Secondo, Lenize da Silva Rodrigues, Leandro Pereira Miranda Ramos, Ana Lívia Carvalho Bovolato, Diego Noé Rodriguez-Sanchez, Marcone Lima Sobreira, Marcelo Padovani de Toledo Moraes, Matheus Bertanha

**Affiliations:** 1 Department of Surgery and Orthopedics, Botucatu Medical School, São Paulo State University—UNESP, Botucatu 18618-687, Brazil; 2 Applied Biotechnology Laboratory, Clinical Hospital of Botucatu Medical School, São Paulo State University—UNESP, Botucatu 18618-687, Brazil; 3 Department of Pathology, Botucatu School of Medicine, São Paulo State University—UNESP, Botucatu 18618-687, Brazil

**Keywords:** models, animal, peripheral arterial disease, tissue engineering, endothelium, mesenchymal stem cells, immune response, cell differentiation

## Abstract

Peripheral arterial disease (PAD) is the main cause of mortality in the western population and requires surgical intervention with the use of vascular substitutes, such as autologous veins or Dacron or PTFE prostheses. When this is not possible, it progresses to limb amputation. For cases where there is no autologous vascular substitute, tissue engineering with the production of neovessels may be a promising option. Previous experimental studies have shown in vitro that rabbit vena cava can be decellularized and serve as a scaffold for receiving mesenchymal stem cells (MSC), with subsequent differentiation into endothelial cells. The current study aimed to evaluate the behavior of a 3D product structure based on decellularized rabbit inferior vena cava (IVC) scaffolds seeded with adipose-tissue-derived stem cells (ASCs) and implanted in rabbits dorsally subcutaneously. We evaluated the induction of the inflammatory response in the animal. We found that stem cells were positive in reducing the inflammatory response induced by the decellularized scaffolds.

## 1. Introduction

The treatment of peripheral arterial disease (PAD) [1] with limb-threatening ischemia is complex, and surgical revascularization is often needed [2,3]. In cases where it is not possible to use an autologous vein for a conventional bypass, synthetic arterial substitutes, such as Dacron^®^ or polytetrafluoroethylene (PTFE) prostheses can be used [4]. The major limitations for the use of synthetic material derive mainly from infections of the prosthesis or at the incision site, as well as the high risk of contamination of the prosthesis from distant infections [3]. In addition, for below-knee prosthetic bypass there is the limitation of poor arterial outflow or incompatibility between caliber and flow [2,4]. Endovascular procedures can be conducted but sometimes have limitations too. When revascularization is indicated but not carried out, the risk of amputation greatly increases [2]. Therefore, the tissue engineering of blood vessels (TEBV) represents a promising perspective to produce personalized vascular substitutes to be used in revascularization surgeries [5]. A major challenge for TEBV to widely reach clinical practice is the production of a functional, resistant, and three-dimensional blood vessel [6,7]. For that, the basis of development requires that the scaffold (synthetic or biological) be sufficient to provide the necessary support for the implanted stem cells to differentiate, renew the extracellular matrix (ECM) and do not induce immunological reactions [8,9,10].

The use of decellularized blood vessels as scaffolds presents some initial biological advantages for TEBV [6,9]. They harbor their own characteristics such as the composition and resistance of the ECM [5,9]. However, for the use of biological material as a scaffold, it is necessary to attain efficient decellularization that guarantees an adequate elimination of cell surface antigens to avoid an immune-mediated rejection of the transplanted tissue and, consequently, the risks of graft loss, including the risk of death or contamination [11,12,13,14].

This study intended to evaluate 3D structures produced by the decellularization of rabbits’ inferior vena cava (IVC) with 1% sodium dodecyl sulfate (SDS) for 2 h (scaffolds), repopulated or not with ASCs (autologous and allogeneic) compared to allogeneic IVCs, implanted in the back of rabbits, maintained for 60 days, to study the inflammatory patterns and the behavior and reactions induced by these structures in the living organism.

## 2. Materials and Methods

### 2.1. Animal Handling Conditions and Tissue Acquisition

A total of 18 adult New Zealand rabbits were used, all nonpregnant females weighing between 2.5 and 3.5 kg. The IVCs were obtained from 6 donor animals, and 2 g of adipose tissue (AT) was also obtained from two of these animals to generate the allogeneic cell bank; one animal was the donor of allogeneic IVC to be used in natura at the time of implantation.

The animals were housed under controlled conditions and fed a standard pellet diet with water ad libitum. Before the surgical procedures to obtain the AT and IVC, the animals were given an intramuscular combination of ketamine 10 mg/kg (Dopalen^®^, 100 mg/mL, Paulínia, SP, Brazil), xylazine hydrochloride 3 mg/mL kg (Anasedan^®^ 2%, 20 mg/mL, Paulínia, SP, Brazil), and acepromazine 0.1 mg/kg (Apromazin^®^ 0.2%, 200 mg/mL, Barueri, SP, Brazil). In addition, a local anesthetic injection, lidocaine 7 mg/kg (Xylocaine 1% 20 mg/mL, Cristália, Butantã, SP, Brazil) was administered at the site designed for the surgical incision. Harvesting was carried out under aseptic conditions and at the end of the procedure, the animals were euthanized with pentobarbital.

### 2.2. Scaffolds Production

The IVCs were sectioned into segments of approximately 2 cm in length, totaling 12 fragments. Decellularization was carried out using the protocol with 1% SDS for 2 h under shaking in a Shaker (News Brunswick Scientific^®^ Edison, NJ, USA), at 37 °C. (Supplementary file S1) [9,13,15]. The fragments were stored in a refrigerator at 4 °C in a sterile solution containing antibiotics and fungicide. 

### 2.3. Obtaining Adipose-Derived Stem Cells (ASCs)

In order to obtain autologous ASCs, the animals were anesthetized, then 2 g of AT was surgically removed from the interscapular region and stored in a conical tube containing a solution of N-2-hydroxyethylpiperazine-N-2-ethanesulfonic acid (HEPES) supplemented with penicillin, 100 mg/mL streptomycin, and 25 mg/mL amphotericin B (2 mmol/L 1-glutamine; Invitrogen™, Waltham, MA, USA). ASCs were obtained by enzymatic dissociation with collagenase type I (Invitrogen™, Waltham, MA, USA). Cell culture procedures were performed with an initial cell count of 6 × 10^4^ cells/cm^2^, obtained from 12 fragments of adipose tissue. These cells were seeded and expanded in 25 cm^2^ culture flasks using Dulbecco’s modified Eagle’s medium (DMEM), supplemented with 10% fetal bovine serum (FBS), 100 U/mL penicillin, 100 mg/mL streptomycin, 25 mg/mL amphotericin B (2 mmol/L of l-glutamine; Invitrogen™, Waltham, MA, USA), 1% (*v/v*) of minimal essential medium (MEM), an essential amino acid solution (Invitrogen™, Waltham, MA, USA), and 0.5% (*v*/*v*) of 10 mM MEM nonessential amino acid solution (Invitrogen™, Waltham, MA, USA) until the number of cells required for the entire study was reached. ASCs were analyzed phenotypically by flow cytometry (FC) using CD45, CD44, CD90, and CD11b, and by tri-lineage differentiation techniques (StemPro™ adipogenesis, chondrogenesis, and osteogenesis differentiation kits; Invitrogen, Waltham, MA, USA). 

### 2.4. Experimental Groups

For all experiments, the vessels (with or without cells) were surgically fixed in the subcutaneous tissue over the dorsal muscular fascia. Four groups were formed (in triplicate)—group 1: implantation of a fragment of allogeneic inferior vena cava in natura (allogeneic IVC); group 2: implantation of a decellularized vein fragment with SDS without the addition of cells (decellularized IVC-SDS); group 3: implantation of a decellularized vein fragment with SDS + 1 × 10^5^ allogeneic ASCs (decellularized IVC-SDS + allogeneic ASCs); group 4: implantation of a decellularized vein fragment with SDS + 1 × 10^5^ autologous ASCs (decellularized IVC-SDS + autologous ASCs). 

### 2.5. Biological Scaffold and ASCs Seeding 

The ASCs (1.3 × 10^5^) were submitted to the cytoplasmic labeling process with Qtracker 605 (CellLabeling Kits—Invitrogen™, Waltham, MA, USA). A solution containing 10 nM of Qdots (1 µL of solution A mixed with 1 µL of solution B, for each 1 × 10^5^ of cells), added in 200 µL of DMEM was used. This solution was homogenized with the cell pellet previously obtained and kept in a 5% CO_2_ incubator at 37 °C for a period of 45 min, protected from light. After the incubation period, the ASCs were washed with D-PBS and diluted in PuraMatrix^®^ peptide hydrogel (BD Biosciences, Franklin Lakes, NJ, USA). A total of 10 µL of this solution was pipetted into the lumens of each scaffold. The experiment was carried out in triplicate, maintained for 2 h in culture for cell preadherence in a DMEM culture medium in a CO_2_ incubator. With a small fragment of the scaffold with ASCs, an immunofluorescence (IF) analysis was performed with Fluoroshield™ with DAPI (Sigma, San Luis, MO, USA) to visualize the ASC nuclei to confirm the beginning of scaffold recellularization.

### 2.6. Surgical Experimentation

For the implantation of the grafts, the animals were anesthetized as previously described, and a surgical aseptic technique was used. A small longitudinal incision, 2 cm in length, was done in the dorsal thoracic region of the animal, dissecting up to the limit of the most superficial fascia of the latissimus dorsi, where the study fragments were fixed (only one fragment per animal) with a simple nonabsorbable stitch of NYLON 4-0 (Ethicon^®^, Somerville, NJ, USA) at each end of the implant, for identification at the time of explantation. The animals were kept in special housing as previously described and monitored daily, being euthanized after the 60-day observation period. The fragments were surgically collected for a histomorphological analysis.

### 2.7. Analysis of Interleukins and Oxidative Stress

During the monitoring period, 1.5 mL of peripheral blood was collected from the marginal ear vein of each animal before the procedure and 1, 7, 14, 30, and 60 days after the surgical procedure. Each sample was centrifuged at 2000× *g* for 5 min to obtain plasma, then kept frozen at −80 °C until inflammatory interleukins (IL6, TNF-α) and anti-inflammatory interleukins (IL-4, IL10) were measured by an enzyme-linked immunosorbent assay (ELISA) (all of them, Invitrogem™, Waltham, MA, USA). At the end of the study, one fragment of the implanted tissue, 0.5 cm in length, was subjected to maceration and the same interleukins were evaluated. 

In addition, analyses were conducted for the detection of oxidative stress by the method of the malonaldehyde (MDA) reaction with thiobarbituric acid (TBARS) [16]. The MDA dosage of the samples was intended to assess oxidative stress variations caused by implants. 

### 2.8. Histomorphology 

The collected fragments (60 days after implantation) were sectioned into two equal parts, one being placed in 10% buffered formaldehyde to make paraffin-embedded blocks, and the other kept frozen for a fresh frozen section stained with Qtracker (red) under a fluorescence microscope, to identify the ASCs, in addition to being macerated later to measure tissue ILs. 

Slides were prepared from the paraffin blocks for standard hematoxylin–eosin (HE) histology and for immunohistochemistry (IHQ) with the analysis of anti-CD31 primary marker diluted 1 in 10 (Novus Biologicals™, Englewood, CO, USA) and marked with secondary antibody Dako EnVision™ + Dual Link System-HRP for the identification of endothelial cells. The slides made with fresh material were destined for a blinded analysis of cell nuclei by immunofluorescence with the 4′,6′-diamino-2-phenyl-indole (DAPI) fluoroshield sealant (Sigma™, Cotia, SP, Brazil). In addition, separate slides with anti-CD31 stained with Fluorescein-5-isothiocyanat (FITC) were made and taken for photomicrographs under a confocal microscope (LEICA™ TSC SP8, Wetzlar, Germany).

### 2.9. Statistical Analysis

The comparison between means at time points was conducted using a repeated measures design to assess the interaction between groups versus time points. In cases where the data presented a symmetrical distribution, an ANOVA for repeated measures was used followed by Tukey’s multiple comparison test adjusted to the design. When the distribution was skewed, a gamma distribution adjustment was conducted for the same design, followed by Wald’s multiple comparison tests. In all tests, the significance level was set at 5%. All analyses were conducted using SAS for Windows, v.9.4 (SAS software for Windows version 9.4, SAS Institute Inc, Cary, NC, USA).

## 3. Results

### 3.1. Animals 

A total of 18 adult New Zealand rabbits were randomly used in all experiments and no one was excluded.

### 3.2. Vein Decellularization, ASC Characterization, and In Vivo Experimentation 

**Figure 1:**
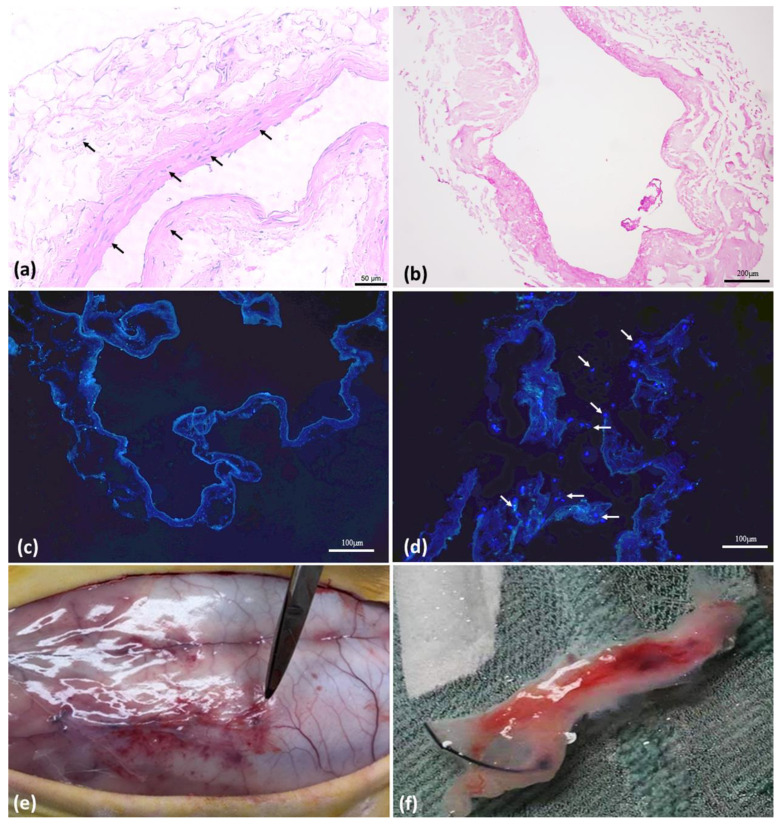
Pre and post implantation tissue analysis. (**a**). Photomicrograph in HE of inferior vena cava (IVC) in natura, magnification: 400×. (**b**) Photomicrograph in HE of decellularized IVC with 1% SDS for 2 h, magnification: 100×. (**c**) Immunofluorescence photomicrograph with DAPI of decellularized IVC with 1% SDS for 2 h, without nuclei marking, magnification: 200×. (**d**) Immunofluorescence photomicrograph with DAPI of decellularized IVC with 1% SDS for 2 h and seeded with 1 × 10^5^ ASC, immediately before implantation, with nuclei marking, magnification: 200×. (**e**) Surgery to harvest the implanted tissue after 60 days. (**f**) Tissue explanted after 60 days.

HE photomicrographs show the comparative result between in natura IVC (Figure 1a) and decellularized IVC (Figure 1b). The IF-DAPI photomicrographs show the comparative result between the decellularized IVC (Figure 1c) and the decellularized IVC with ASCs seeding in the proposed model before implantation (Figure 1d).

ASCs were analyzed for phenotypic characterization by FC. The cell surface markers CD44 and CD90 showed peaks of positive events, while the hematopoietic and immunological cell markers CD45 and CD11b, respectively, were negative, confirming the cell phenotype. The trilineage differentiation challenge was positive, completing the protocol for stem cell confirmation.

The implants were performed as previously described; blood was collected for cytokine analysis and after 60 days of implantation, the animals were euthanized, and the samples were collected (Figure 1e,f).

### 3.3. Interleukin Analysis

#### 3.3.1. Plasma

**Figure 2:**
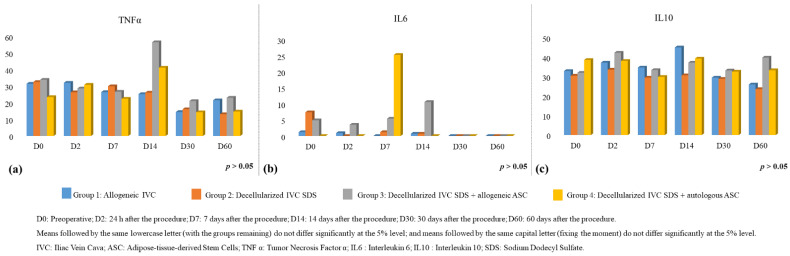
Analysis of plasma cytokines by Elisa. (**a**) Analysis of Tumor Necrosis Factor α (TNF α); (**b**) Analysis interleukin 6 (IL6); (**c**) Analysis of interleukin 10 (IL10).

The study of serum levels of ILs showed statistically similar serum levels between the groups studied for all moments, with IL4 being the only one with a result equal to zero in all analyses, being considered not detectable under the conditions of this experiment (Figure 2).

#### 3.3.2. Frozen Explant Tissue Homogenate

**Figure 3:**
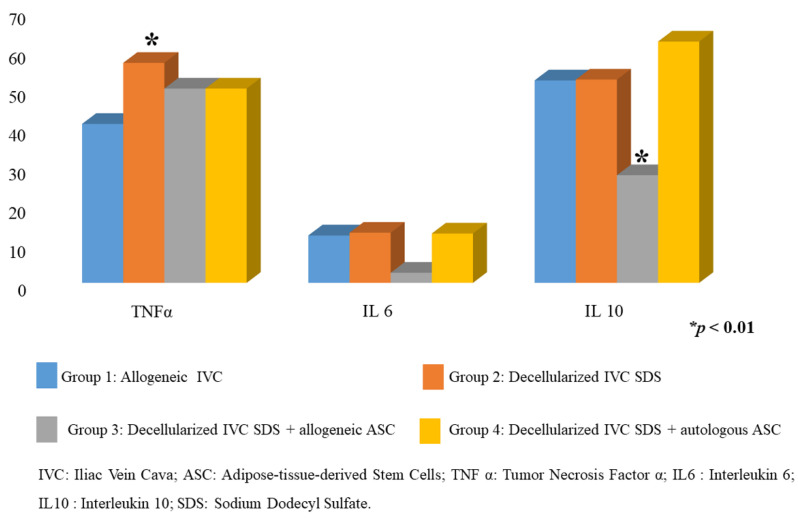
Tissue interleukin analysis (Tumor Necrosis Factor α (TNF α), interleukin 6 (IL6), and interleukin 10 (IL10).

The analysis of ILs in frozen explant tissue homogenate at 60 days showed a result for IL4 equal to zero in all samples, considered as not detectable under the conditions of this experiment. The results for TNFα were higher for group 2 (decellularized vein), which meant a higher inflammatory response compared to the other groups which presented lower measurements (*p* < 0.01). For the values measured by the dosage of IL6, there was no statistical difference between the groups; therefore, it was not possible to use this parameter to differentiate the inflammation caused by the implants under the conditions of this experiment. For IL10, only group 3 (decellularized IVC-SDS + allogeneic ASC) was found to have a lower dose compared to the other groups, with a statistically significant difference (*p* < 0.01), suggesting a decrease in the regulation of the inflammatory IL10-mediated process in this group (Figure 3).

#### 3.3.3. Oxidative Stress by Plasma MDA Analysis 

**Figure 4:**
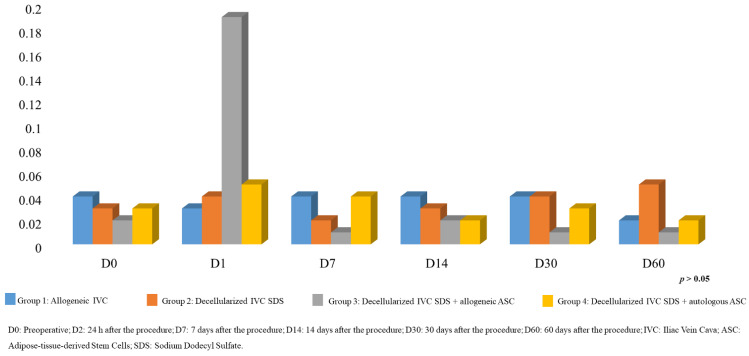
Malonaldehyde (MDA) analysis by moments. D0: Preoperative; D2:24 h after the procedure; D7: 7 days after the procedure; D14: 14 days after the procedure; D30: 30 days after the procedure; D60: 60 days after the procedure.

Figure 4 shows the results of the oxidative stress analyses at all moments of the study. In general, it was not possible to find statistical differences in the serum MDA analysis in plasma; therefore, no inference could be made regarding the influence of the procedures adopted in this study and the increase or decrease in oxidative stress.

### 3.4. Histomorphology

**Figure 5:**
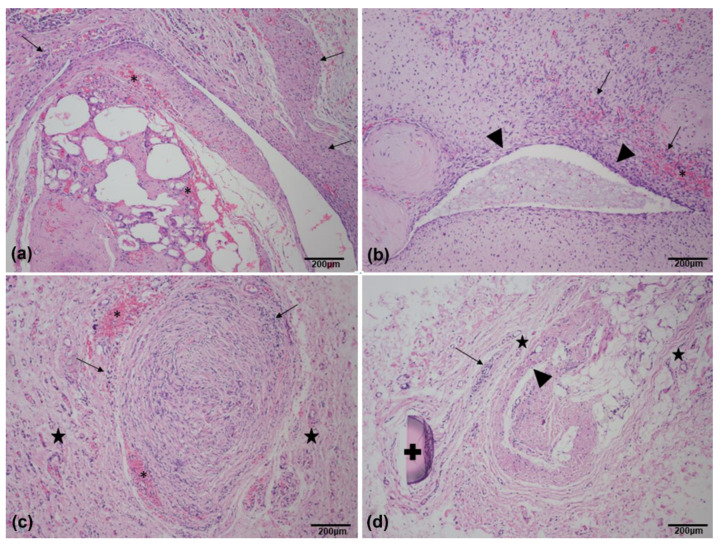
Photomicrography in HE, magnification: 100×. Scaffold explants at 60 days. (**a**) Group 1—allogeneic IVC showing inflammatory cells (lymphocytes, plasma cells, and histiocytes), persistence of native vein structures (endothelium) (arrows), and hemorrhage (asterisk); (**b**) group 2—decellularized IVC-SDS showing small endothelial neoformation, immature fibroblasts (myofibroblasts) (arrowhead), evidence of inflammatory reaction (arrows), and hemorrhage (asterisk); (**c**) group 3—decellularized IVC-SDS + allogeneic ASCs showing vascular proliferation (star), low inflammatory reaction (arrows), fibroblasts (myofibroblasts) proliferation (arrows), and hemorrhage (asterisk); (**d**) group 4—decellularized IVC-SDS + autologous ASCs showing greater adjacent vascular neoformation (star), endothelium partially repopulating (arrowhead), and significantly less inflammatory reaction (arrows), except at the region of the NYLON stitch (plus sign).

Analysis in HE of the fragments implanted for 60 days showed some endothelization, with good cell colonization in all groups. However, some qualitative differences were observed with the same regularity in each group, such as the presence of a greater inflammatory response in group 1—allogeneic IVC, due to the presence of lymphocytes, plasma cells, and histiocytes, in addition to the persistence of native vein structures, the same endothelium, and hemorrhage; for group 2—decellularized IVC-SDS, small endothelial neoformation and immature fibroblasts (myofibroblasts), inflammatory reaction, and hemorrhage were seen; in group 3—decellularized IVC-SDS + allogenic ASC, vascular proliferation, low inflammatory reaction, fibroblasts proliferation (myofibroblasts), and hemorrhage were observed; Unlike the others, Group 4—decellularized IVC-SDS + autologous ASCs showed greater adjacent vascular neoformation, an endothelium partially repopulating the lumen of the venous scaffold, and significantly less inflammatory reaction, except at the region of the NYLON stitch (Figure 5).

**Figure 6:**
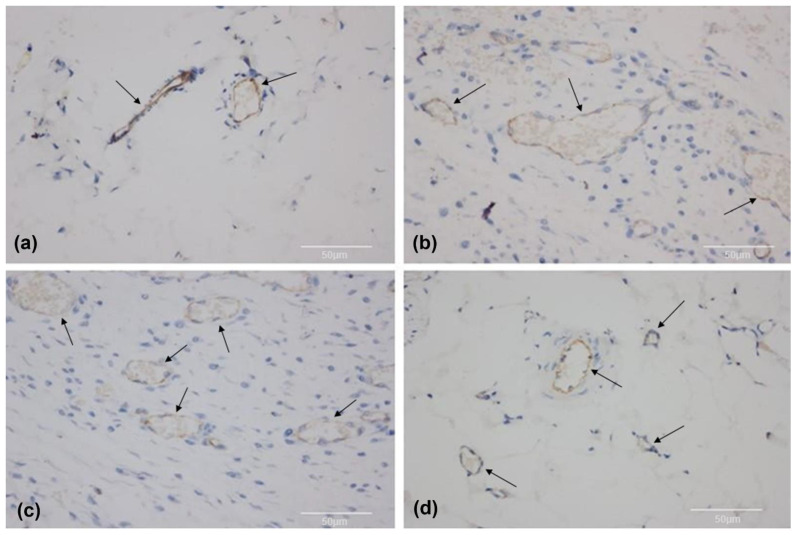
Immunohistochemistry, slide analysis under light microscopy with CD31 endothelium marker antibody (arrows); magnification: 400×. (**a**) Group 1; (**b**) group 2; (**c**) group 3; (**d**) group 4.

In the immunohistochemical analysis, the CD31 antibody, a vascular endothelium marker, was present in all groups; however, it occurred in slightly different patterns between the groups, corresponding to the histological findings of using HE, with a lower intensity of endothelial staining for group 1 and a less organized pattern for groups 2 and 3 as compared to group 4 (Figure 6).

**Figure 7:**
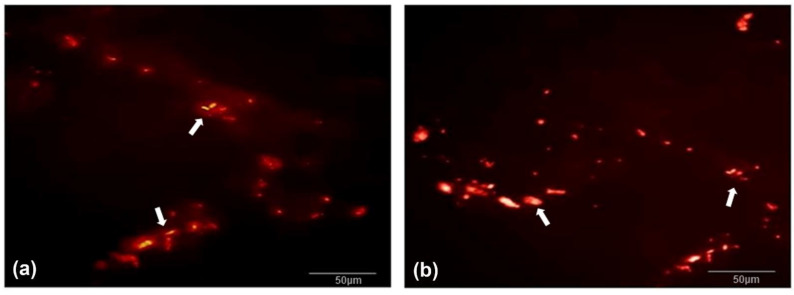
Analysis in fluorescence microscopy; magnification: 400×. (**a**) Group 3, (**b**) group 4, showing cell marking signals (ASC) with Qtracker (red fluorescence in nanocrystals deposited in the cytoplasm) (white arrows).

**Figure 8:**
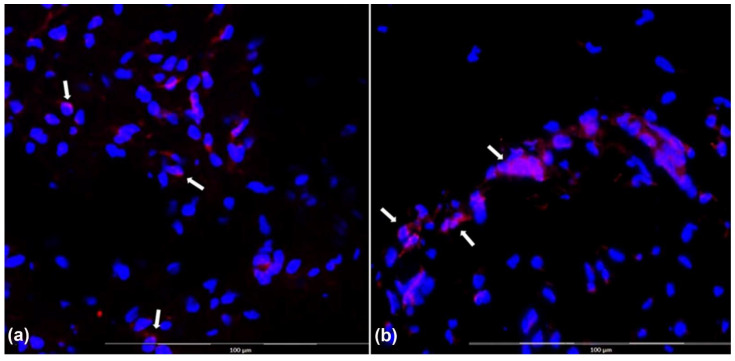
Immunofluorescence on frozen tissue; magnification: 200×. (**a**) Decellularized veins with allogeneic ASCs, cell nuclei in blue (DAPI) and red, and cytoplasm of tissue tagged ASCs (Qtracker); (**b**) decellularized veins with autologous ASCs, cell nuclei in blue (DAPI) and red, and cytoplasm of tagged ASCs in tissue (Qtracker) (arrows point to tagged cells) (white arrows).

Immunofluorescence to evaluate Qtracker-labeled ASCs (in red) in groups 3 and 4 (Figure 7) with overlabeling with DAPI showed that there were cells labeled with both markers in the two groups where this was possible, suggesting that they came from the ASC culture (groups 3 and 4) (Figure 8).

**Figure 9:**
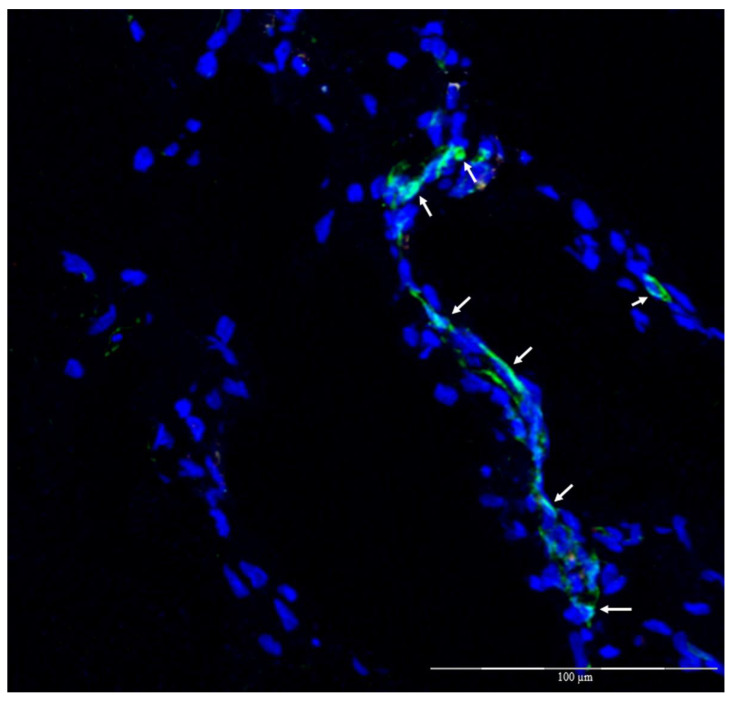
Immunofluorescence on frozen tissue; magnification: 200×. Decellularized veins with autologous ASCs, cell nuclei in blue (DAPI), and green, and cytoplasmic membrane labeled with anti-CD31 (FITC), only for group 4 (arrows point to labeled cells) (white arrows).

Another analysis by immunofluorescence was conducted with CD31 + FITC (green) and DAPI (blue), in addition to the previous staining with Qtracker (red) and it was possible to see, in confocal fluorescence microscopy, the occurrence of some endothelial cells that originated from the implanted culture since they presented the three markings simultaneously only in group 4 (Figure 9).

## 4. Discussion

The conventional treatment for PAD is clinical, aiming at the control of risk factors. In a situation of critical ischemia, however, the alternatives are endovascular treatments with angioplasty or revascularization surgeries (bridges/bypass using an autologous vein or synthetic material) [2]. However, for a portion of patients, revascularization treatments are not applied, increasing the risk for limb amputations [4]. Consequently, biotechnology becomes a partner for the development of biomimetic products, and numerous studies have pointed to the possibility of the production of organs or tissues that may be promising for the regeneration or replacement of damaged vessels, considering that there is already enough technology for the differentiation of ASCs into endothelium, smooth muscle, and other tissues [17,18,19,20].

In a review article, Afra et al. [21] compared the results of preclinical research with the use of autologous or allogeneic ASCs for the development of blood vessels and concluded that autologous cells are preferred. Our work goes in the same direction, pointing out the advantages of the use of autologous cells. In that same review, the authors studied the potential of ASCs in engineering functional blood vessels compared to other cell types, such as bone marrow mononuclear cells, endothelium precursor cells, adult autologous smooth muscle cells, autologous endothelial cells, embryonic stem cells, and pluripotent stems [21]. It was concluded that ASCs were still the preferred cells for tissue engineering of blood vessels, mainly because of their multipotent potential and ease of obtaining them in quantity. These reasons also guided our work. ASCs can also secrete a broad spectrum of bioactive macromolecules that help the regeneration of injured tissues and have immunomodulatory properties [21].

The most suitable scaffolds for tissue engineering of blood vessels should be able to withstand vascular stress without degeneration, do not promote thrombotic events, present good interaction with adjacent tissues and implanted cells, and enable nutrient transport through the porosity of their wall until they are fully developed [20]. More than one of our previous studies [15] have determined that the method used in the production of a biological scaffold produced by the decellularization of the vena cava in the rabbit model meets these requirements, and the current study demonstrates that the interaction with autologous ASCs promotes a favorable environment, generating the ideal adaptive conditions when it is implanted on the animal’s back.

The immune response triggered by the implantation of scaffolds in the in vivo animal model varied between groups and according to the form of analysis. For the evaluation of serum results, no significant differences were found between the groups at the times analyzed, which could be explained by the volatility of the production of these cytokines after the first hours of surgery, the small aggression caused by the surgery, and by the small amount of tissue implanted. For the analysis of the homogenate of part of the tissue collected 60 days after implantation, unlike the serum analysis, there was some difference between the groups even in the latest period assessed. A greater proinflammatory reaction, mediated by TNFα, was found in group 2, which contained only decellularized tissue compared to the other groups, but this was not seen in the assessment of IL6. Such finding is expected since the scaffold basically behaves like a foreign body [22]. However, a decrease in the IL10-mediated anti-inflammatory action was seen in the animals of group 3, which received allogeneic ASCs, which may be a sign of a delayed rejection reaction [23,24]. 

Interestingly, our experiments did not detect serum or tissue IL4 in any group. IL4 was previously selected to assess whether ASCs would have any anti-inflammatory activity in the experiment with biological scaffolds. However, we could not find significant levels of IL4 in any of our experiments; perhaps this can be explained by the volatility of the cytokine in the samples, the sensitivity level of the test, or the animal model used, or technical problems in the experiment; however, this question remains open [25]. Regardless, the immunomodulatory role of ASCs has already been established in experiments of this nature, and it is likely that they perform this function while not differentiating in other cell types [26,27,28,29].

Regarding the cellular differentiation of ASCs and the structural organization of these neovessels, the implants of groups 3 and 4, composed of decellularized vena cava + ASCs, were more promising. The presence of these red-marked cells, derived from viable cultures, was detected in the tissue even after 60 days of implantation.

## 5. Conclusions

The group with a decellularized vena cava scaffold (SDS) recellularized with autologous ASCs showed a lower host immunogenic response considering serum and tissue cytokines. This group also showed the best histological analyses, with a greater cellular organization and endothelial differentiation compared to using allogeneic tissue, unpopulated scaffolds, or allogeneic-ASCs-repopulated scaffolds.

## Data Availability

All survey data can be requested from the corresponding author.

## Supplementary Materials

The following supporting information can be downloaded at: https://www.mdpi.com/article/10.3390/biom12121776/s1, Suplementary S1: Decellularization protocol.
