# Supplementary material for: Evaluation of Biointegration and Inflammatory Response to Blood Vessels Produced by Tissue Engineering—Experimental Model in Rabbits
Source: Biomolecules. 2022 Nov 29;12(12):1776. doi: 10.3390/biom12121776 (PMC9775166; doi:10.3390/biom12121776)
Supplement: Supplementary file 1 [file biomolecules-12-01776-s001.zip › biomolecules-1956716-supplementary.pdf]

Suplementary S1

Vein decellularization protocol

Material

- Conical tube
- Injection water
- Sodium duodecyl sulfate (SDS) 1%
- Clean and fragmented sample

Solution preparation Sodium duodecyl sulfate (SDS) 1%: 200mL of ultrafiltered H20 + 2.5g SDS (molecular weight 288.38, concentration 0.35 M, Sigma Aldrich, San Luis, Missouri, USA)

Ps. Each conical tube must be filled with 25 mL of the 1% SDS solution.

Procedure

In a laminar flow chamber, sterile environment, the samples (veins) must go through the cleaning procedure to ensure that there are no remains of blood and fat compound, then separated into fragments according to the study protocol. The fragmented samples will be stored at -80ºC until the moment of decellularization.

Before the decellularization process, the fragments will be thawed at room temperature, washed with saline solution, and stored 5 samples per conical tube containing 25mL of 1% SDS solution, seal the lid with parafilm to prevent leakage. Place the tubes in a horizontal position on the shaker tray and fix them with tape, keep stirring at 1200rpm for 2 hours at 37ºC (Shaker News Brunswick Scientific® with the controlled temperature at 37ºC). The fragments went through a cycle of 3 washes with sterile saline solution and were conditioned and preserved in a refrigerator at 4ºC in a sterile solution containing antibiotic and antifungal until the moment of use in culture.

Observations: Use mask for handling SDS, mucosa irritant substance.
